# Fabrication of Lead Free Borate Glasses Modified by Bismuth Oxide for Gamma Ray Protection Applications

**DOI:** 10.3390/ma15030789

**Published:** 2022-01-21

**Authors:** Yas Al-Hadeethi, M. I. Sayyed, Abeer Z. Barasheed, Moustafa Ahmed, Mohamed Elsafi

**Affiliations:** 1 Department of Physics, Faculty of Science, King Abdulaziz University, Jeddah 21589, Saudi Arabia; abeer.barasheed@gmail.com (A.Z.B.); oahafidh@kau.edu.sa (M.A.); 2 Lithography in Devices Fabrication and Development Research Group, Deanship of Scientific Research, King Abdulaziz University, Jeddah 21589, Saudi Arabia; 3 Department of Physics, Faculty of Science, Isra University, Amman 11622, Jordan; mohammed.alsyyed@iu.edu.jo; 4 Department of Nuclear Medicine Research, Institute for Research and Medical Consultations (IRMC), Imam Abdulrahman bin Faisal University (IAU), Dammam 31441, Saudi Arabia; 5 Physics Department, Faculty of Science, Alexandria University, Alexandria 21511, Egypt; mohamedelsafi68@gmail.com

**Keywords:** radiation shielding glasses, narrow beam technique, NaI(Tl) scintillation detector, ionizing radiation

## Abstract

In the present work, bismuth borate glass samples with the composition of (99-*x*) B_2_O_3_ + 1Cr_2_O_3_ + (*x*) Bi_2_O_3_ (*x* = 0, 5, 10, 15, 20, and 25 wt %) were prepared using the melt quenching technique. The mass attenuation coefficient (MAC) of the prepared glass samples was measured through a narrow beam technique using a NaI(Tl) scintillation detector. Four point sources were used (^241^Am, ^133^Ba, ^152^Eu, and ^137^Cs) to measure the MAC for the prepared glasses. The experimental data were compared with the theoretical results obtained from the XCOM, and it was shown that for all samples at all tested energies, the relative deviation between the samples is less than 3%. This finding signifies that the experimental data can adequately be used to evaluate the shielding ability of the glasses. The MAC of the sample with *x* = 25 wt % was compared with different lead borate glasses and the results indicated that the present sample has high attenuation which is very close to commercial lead borate glasses. We determined the transmission factor (TF), and found that it is small at low energies and increases as the energy increases. The addition of Bi_2_O_3_ leads to reduction in the TF values, which improves the shielding performance of the glass system. The half value layer (HVL) of the BCrBi-10 sample was 0.400 cm at 0.595 MeV, 1.619 cm at 0.2447 MeV, and 4.946 cm at 1.4080 MeV. Meanwhile, the HVL of the BCrBi-20 sample is equal to 0.171 and 4.334 cm at 0.0595 and 1.4080 MeV, respectively. The HVL data emphasize that higher energy photons tend to penetrate through the glasses with greater ease than lower energy photons. Furthermore, the fast neutron removable cross section (FNRC) was determined for the present samples and compared with lead borate glass and concrete, and the results showed a remarkable superiority of the bismuth borate glass samples.

## 1. Introduction

The ionizing radiation in the human environment that results from normal background radioactivity, mining, milling, use of synthetic radioactive isotopes in nuclear power, nuclear research, space research, etc., all cause human exposure to the hazards of ionizing radiation. The scientific trend towards replacing fossil fuels by generating energy from nuclear reactors, and the accompanying possibilities of radioactive leakage and dangerous radioactive waste, which pose great risks to humans, requires serious research and effective means of protection and shielding [1,2]. These means should take into account the dose of radiation that the population may be exposed to in the event of a nuclear accident.

This protection can be achieved through the use of effective shields, which requires the studying of the characteristics of potential shielding materials, such as mechanical properties, characteristics of different radiation attenuation, expected energies, cost and availability of materials, ease of production, effectiveness, and the likelihood of low cost. Another critical property is the extent of the shielding material’s resistance to damage that may be caused as a result of exposure to ionizing radiation, in addition to making sure that those shields are not toxic. It is also required that the effective shielding material causes significantly higher intensity attenuation of the incident radiation within a small penetration length (thickness), as well as a high radiation-absorption cross-section. Furthermore, the effects of radiation on the optical and mechanical features of the shielding material must be minimal [3].

Accordingly, the development of radiation protection mediums has been constantly fascinating in the fields of medical, industrial, agricultural, near future power generation fusion reactors, etc. Concrete, lead, and some high-Z materials are promising to protect against radiation. However, the shielding against a mixture of gamma and neutrons demands to investigate an optimal composition of low- and High-Z elements. Traditionally, steel, lead, and concrete are utilized as protection from a number of ionizing radiation sources. In the present work, we prepare and explore the radiation shielding properties of B_2_O_3_-Bi_2_O_3_- bismuth borate glasses due to their unique features.

Several considerations will be taken into account when developing our glass samples; namely, they should acquire high transparency to the visible part of the electromagnetic spectrum, and high mass density in order to ensure effective shielding properties and assure that the interaction probability between the photons and glass is significantly high. This high interaction probability, accordingly, indicates that the ionizing radiation energy will be significantly attenuated and the photons lose their capability to penetrate the glass. Increasing the glass density is one of the approaches to enhance the radiation attenuation [4]. Borate-based glasses are well-known for their minimal viscosity, both high chemical durability and transparency to visible light, small glass transition temperature, excellent mechanical stability, and can be acquired cheaply [5,6,7]. These properties made the borate-based glasses as materials of consideration for a variety of applications, including industrial, biomedical, shielding, and many other applications.

In the sector of radiation protection, borate-based glasses are of significant interest as a radiation shielding material. The capability for modifying the density of borate-based glasses could enhance their shielding characteristics. One of the simplest methods for increasing the glass density is incorporating heavy rare-earth oxides (HREO) and heavy metal oxides (HMO) into the glass samples [7,8,9]. Glasses based on heavy metal oxide, such as Bi_2_O_3_, have several utilizations, particularly in glass ceramics, and recently in developing ionizing radiation shielding materials [10,11,12].

Our interest in investigating bismuth borate glasses is attributed to the fact that borate glasses are attractive optical materials with high thermal stability, a significantly high degree of transparency, and a low melting point [13,14]. Moreover, glasses which contain heavy metal oxide (Bi_2_O_3_) have been thoroughly inspected, due to their interesting uses in new technology. Pure Bi_2_O_3_ glass is not a classical glass former, due to both its minute field strength and high polarizability. Consequently, it is not possible to compare it with pure B_2_O_3_ glass [14].

Bi_2_O_3_ is not a classical glass former, owing to the high polarizability and the small field strength of Bi^3+^ ions. In the presence of a glass former, such as B_2_O_3_, SiO_2_, etc., it might build a glass network of BiO_n_ (*n* = 3, 6) pyramids. Hence, due to its double features, being a modifier having [BiO_6_] octahedral and being glass former having [BiO_3_] pyramidal units, bismuth ions might have a significant effect on the electrical characteristics of oxide glasses [15].

Furthermore, borate glasses that contain Bi_2_O_3_ acquired special interest due to their long infrared cutoff and high third order nonlinear optical susceptibility. These special characteristics make them excellent materials for several applications, particularly for developing internal transmission components, ultrafast switches for femtosecond lasers, and high-speed optical data processing systems [16,17,18].

In this work, we aimed to use a melt quenching technique to prepare glass samples of compositions (99−*x*)B_2_O_3_ + 1Cr_2_O_3_ + (*x*)Bi_2_O_3_ (*x* = 0, 5, 10, 15, 20 and 25 wt %). The mass attenuation coefficient (MAC) of the prepared glass samples was measured through a narrow beam technique using a NaI(Tl) scintillation detector.

## 2. Materials and Method

**Figure 1:**
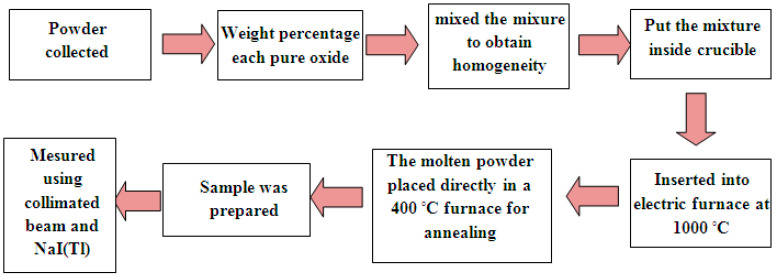
The steps of sample preparation in the present study.

In this work, glass samples with composition (99-*x*)B_2_O_3_ + 1Cr_2_O_3_ + (*x*)Bi_2_O_3_ (*x* = 0, 5, 10, 15, 20, and 25 wt %) were prepared using the melt quenching technique. Equivalent quantities of high purity chemicals (99.8%) B_2_O_3_, Cr_2_O_3,_ and Bi2O3 were taken as chemical intermediates to manufacture this glass system. They were purchased from the Elgamhoria Chemical Company in Egypt. The reason for choosing this glass system is that the boron oxide (B_2_O_3_) is an excellent absorber for neutrons, and Cr_2_O_3_ improves the mechanical, thermal, and optical properties, as well as giving a transparent light green color which adds to the applications in the radiological field. Additionally, Bi_2_O_3_ increases the density of the sample to use as a shield for photons. To reach homogeneity, chemicals were mixed with agate slurry. The mixture was taken into a crucible (made of German refractory bricks) and placed in a 1000 °C electric furnace. The molten mixture was placed in another electronic furnace for annealing at 400 °C for 2 h, with the temperature slowly lowered to remove cracks and thermal strain for the selected glass samples. The steps of manufacturing this glass are summarized in Figure 1. Due to the potential for bremsstrahlung X-ray produced from the interaction of beta particles with high *Z_eff_* materials, samples that do not exceed 25 weight% Bi_2_O_3_ were prepared in the glass system.

**Figure 2:**
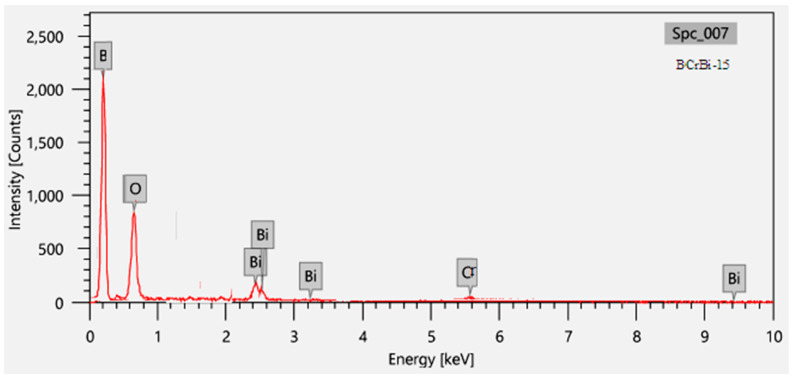
The energy dispersive X-ray analysis (EDX) for BCrBi-15 glass sample.

The chemical compositions of the selected glass system were tabulated in Table 1. To ensure the correct compositions after preparation, the samples were analyzed by EDXor, Energy Dispersive X-ray analysis of the analytical scanning electron microscope (JSM-5300, JEOL) as shown in Figure 2—for example the BCrBi-15 sample, which gives the proportion of each element in the sample [19,20]. The analyses were performed for the sample more than once at different points and then the average was taken for the required percentage. The density was measured, as listed in Table 1, using the Archimedes’ rule. Some of the attenuating properties of photons and neutrons were experimentally studied and compared theoretically with the XCOM program [21,22]. The use of this glass as a protector and a transparent shield for neutrons and photons was examined at the same time.

**Figure 3:**
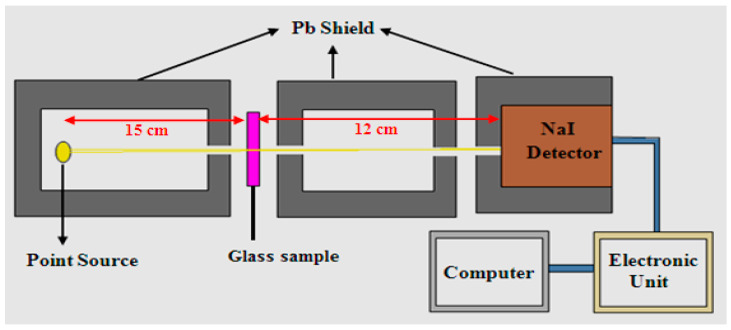
The schematic diagram of the experimental setup of the narrow beam method.

The mass attenuation coefficient (MAC) of the present glass samples was measured through a narrow beam technique. The NaI(Tl) scintillation detector was used [21,22,23,24]. The illustration of this detector is shown in Figure 3. The Genie 2000 software was used to analyze the output spectrum, which is a comprehensive set of capabilities for acquiring and analyzing spectra. First, the detector was calibrated using two point sources (^137^Cs and ^60^Co). Before any measurement, the calibration process of the detector (energy and efficiency calibration) is carried out, and then a substance with a known attenuation coefficient, such as lead, was measured, which was followed by validating the experimental results. Four point sources were used for the measurements to cover the required energy (^241^Am, ^133^Ba, ^152^Eu, and ^137^Cs), and the specification of these sources, as well as its figures, were tabulated in Table 2.

**Figure 4:**
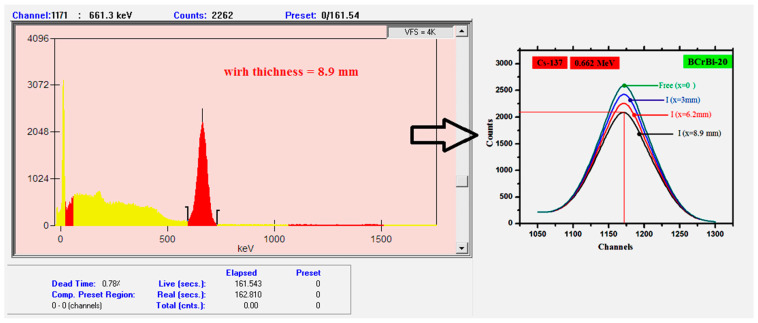
The incident and transmitted spectra for Cs-137 at different thicknesses of the BCrBi-20 glass sample.

The MAC was evaluated using the next equation [25]:

(1) $$MAC=\frac{-1}{d}\ln\left(\frac{I}{I_{0}}\right)$$

where I and *I*_0_ are the intensity or the net count rates in the presence and absence of the studied glass sample, respectively, *d* (g/cm^2^) is the mass thickness (*d* = ρ*x*) with *ρ* (g/cm^3^) being the density of the sample, and *x* (cm) is the thickness of the sample. Figure 4 shows the incident and transmitted spectra for three different BCrBi-20 glass samples with different thicknesses at the same energy (0.662 MeV).

The relative deviation between the experimental and *XCOM* results was calculated from the following equation [26]:

(2) $$dev=\frac{{\left(MAC\right)}_{XCOM}-{\left(MAC\right)}_{Expt.}}{{\left(MAC\right)}_{XCOM}}\times 100\;$$

where (*MAC*)*_XCOM_* and (*MAC*)*_Expt_* are the theoretical and experimental mass attenuation coefficients, respectively. The uncertainty of the experimental results of *MAC* was calculated according to the following equation:

(3) $$\sigma_{MAC}=MAC\cdot \sqrt{{\left(\frac{\partial MAC}{\partial N}\right)}^{2}\cdot \sigma_{N}{}^{2}+{\left(\frac{\partial MAC}{\partial d}\right)}^{2}\cdot \sigma_{d}{}^{2}}$$

where $\sigma_{N}$ and $\sigma_{d}$ are the uncertainty in the count rate and mass thickness of the measured sample, respectively. The linear attenuation coefficient (*LAC*) is a very important parameter to evaluate *HVL*, where the experimental *LAC* can be estimated from (*LAC* = *MAC* × ρ). The *HVL* was determined from the next equation [27]:

(4) $$HVL=\frac{\ln2}{LAC}\;\;\;\;\;\;\;\;\;\;\;\;\;$$

It is also important to calculate the efficiency of this manufactured system in attenuation or shielding at different energies. The radiation protection efficiency (*RPE*) was calculated from the following relationship [28]:

(5) $$RPE\left(\%\right)=\left|1-\frac{I}{I_{0}}\right|\times 100$$

Finally, the effective atomic number (*Z_eff_*) has a positive relationship to understanding and interpreting the results of the attenuation coefficient. *Z_eff_* is given by the following equation [29]:

(6) $$Z_{eff}=\frac{\sum_{i}f_{i}A_{i}{\left(MAC\right)}_{i}}{\sum_{j}\frac{A_{j}}{Z_{j}}{\left(MAC\right)}_{j}}$$

where *f_i_, A_i_*, and *Z_i_* refer to the mole fraction, atomic weight, and atomic number of the *i*^th^ constituent element in the selected glass sample, respectively.

A final term to be discussed is the “effective neutron removal” (R), which refers to the process of removing neutrons from matter in time. At a defined and specific cross-section, the total amount of nuclear energy generated in a fission event from the first (or fast) neutron is equal to the average expected amount of nuclear energy generated in a fission event. Therefore, the fast neutron removal cross section (∑R) values for a substance can be calculated using the following equation:

(7) $$\sum R/\rho =\sum w_{i\;}\left(R/\rho \right)$$

where ∑R/ρ (cm^2^·g^−1^) is the mass removal cross section and *w_i_* is the partial density of the *i*th element (g/cm^3^).

## 3. Results and Discussion

**Figure 5:**
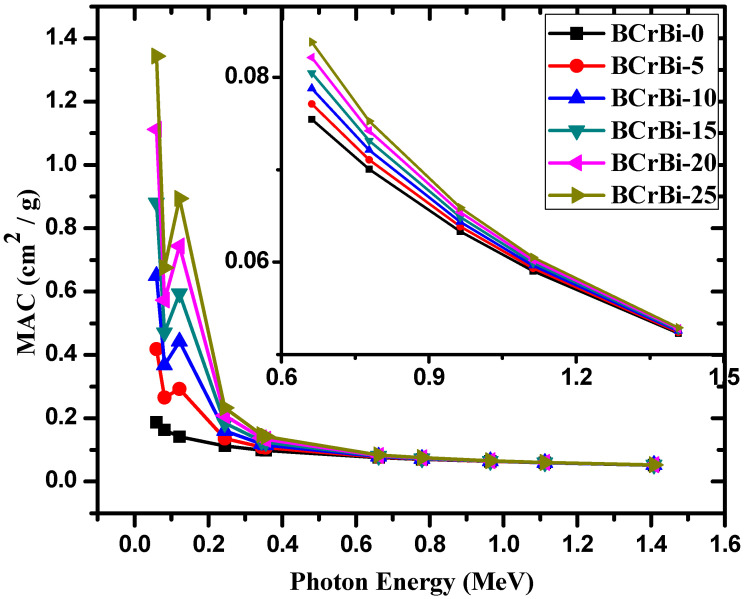
The MAC as a function of energy for the BCrBi glass samples.

The mass attenuation coefficients (MACs) of the BCrBi glass samples were experimentally measured using various radioisotopes at 11 energies ranging from 0.0595 MeV to 1.408 MeV. The experimental data were compared with the theoretical results obtained from the XCOM, and both results are listed in Table 3 and plotted in Figure 5. The relative difference between the experimental and theoretical MAC values are also tabulated in Table 3. For all the samples at all tested energies, the relative deviation between the samples is less than 3%, which confirms the experimental setup used in this work, and signifies that the experimental data can adequately be used to evaluate the shielding ability of the glasses.

**Figure 6:**
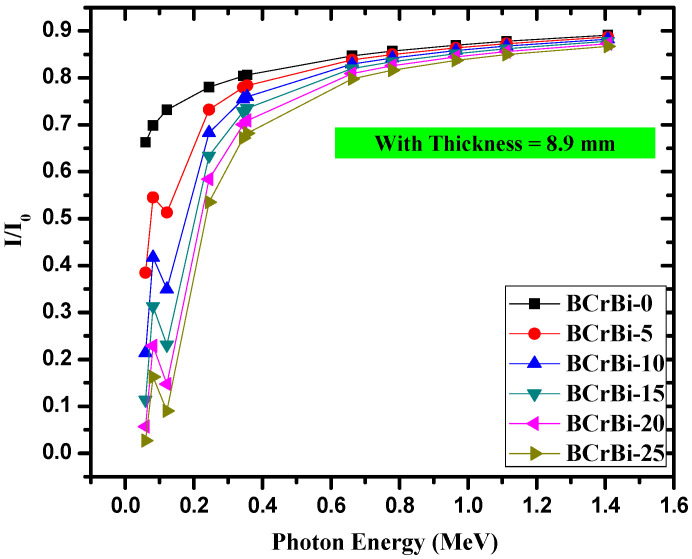
The transmission factor for the BCrBi glass samples.

Figure 6 demonstrates the ratio between the intensity of the photons that pass through the glass and the total photons that are emitted, *I*/*I*_0_, known as “the transmission factor (TF)”. TF provides insight into the glass’s performance as a radiation shield, because if the ratio is in close unity, this means that most of the incoming radiation can pass through the glass. In other words, the glass is not effective in attenuating radiation. On the other hand, if the transmission factor is small, then the intensity I must be much smaller than *I_0_*, which means that most of the photons are stopped by the glass. Thus, the goal is to find a glass with a small TF. From Figure 6, it can be seen that the transmission factor is small at low energies and increases as the energy increases. It can also be said that the attenuation ability of the glasses is high at low energies, and reduces with increasing the energy. TF is very small at low energies for all samples except BCrBi-0, since this glass does not contain any Bi_2_O_3_, causing it to have relatively high TF values. Since Bi has a high atomic number, the addition of Bi_2_O_3_ leads to reduction in the TF values, which improves the shielding performance of the glass system. Naturally, BCrBi-25 has the lowest transmission factor due to the high amount of Bi_2_O_3_ in its composition. At energy of 0.0810 MeV, BCrBi-25 has TF of 0.130, while at 1.1120 MeV, TF is equal to 0.833. Altogether, the figure reveals that the glasses are more effective at low photon energies, and the BCrBi25 glass sample has the best shielding performance because of its high Bi_2_O_3_ content.

**Figure 7:**
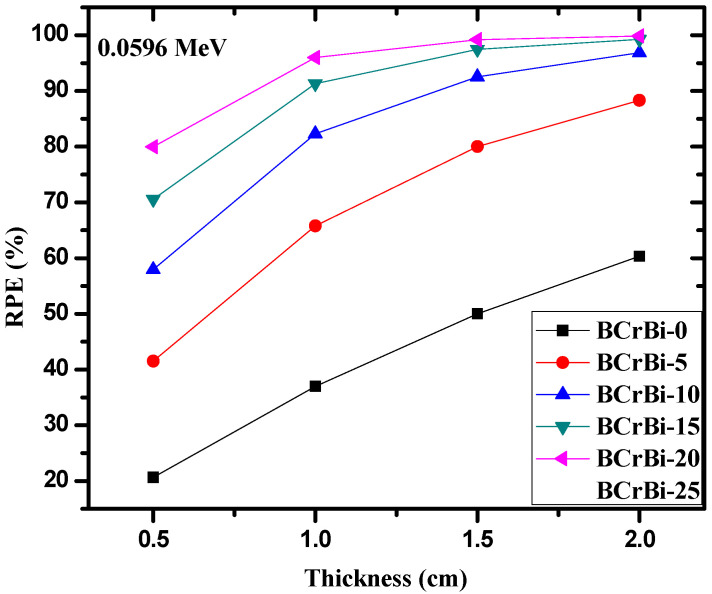
The radiation protection efficiency as a function of glass thickness at 0.0596 MeV.

**Figure 8:**
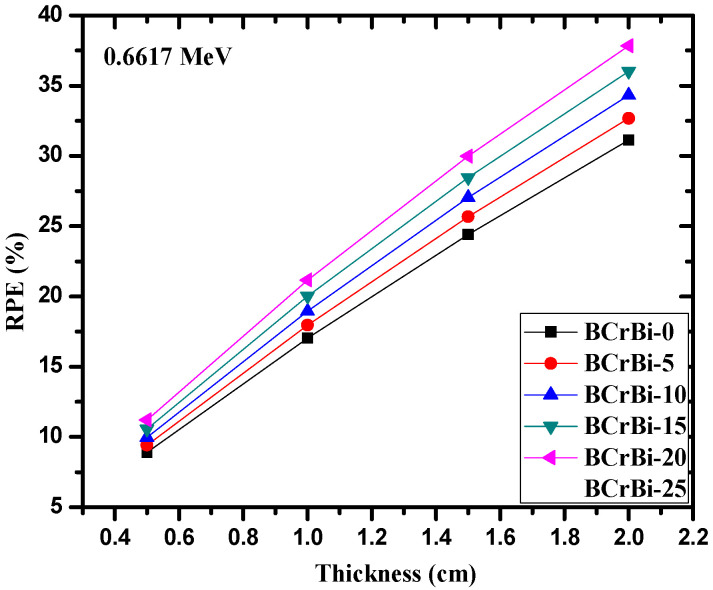
The radiation protection efficiency as a function of glass thickness at 0.6617 MeV.

In Figure 7 and Figure 8, we plotted RPE versus the glass thickness at photon energies of 0.0596 and 0.6617 MeV, respectively. In both figures, RPE increases with the increase in the thickness. This can be explained according to the fact that the probability of photon-glass interaction increases with the increase in the thickness of the glass, where more photons are attenuated by the thick glass sample. This suggests that one effective way to enhance the shielding ability of the samples is to increase the glass thickness. Additionally, we can observe that the RPE values at low energies depend highly on the composition of the glasses, since the difference between RPE and the BCrBi-X is very notable. At higher energies, the composition of the glasses has a weak role on the RPE values and thus on the attenuation performance of the glasses.

**Figure 9:**
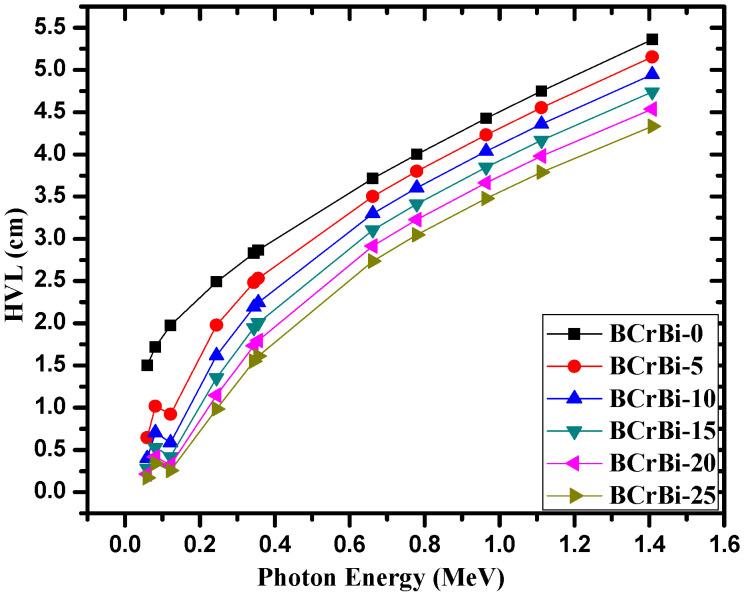
The half value layer of the BCrBiX glasses.

Figure 9 shows the half value layer, or HVL, of the BCrBiX glasses as a function of photon energy. As a general trend, HVL increases as the energy increases. For instance, the HVL of the BCrBi-10 sample increases from 0.400 cm at 0.0595 MeV, to 1.619 cm at 0.2447 MeV, 2.248 cm at 0.3560 MeV, 3.603 cm at 0.7789 MeV, and to 4.946 cm at 1.4080 MeV. Meanwhile, the HVL of the BCrBi-20 sample is equal to 0.171, 0.986, 1.611, 3.047, and 4.334 cm, at the same respective energies. This direct trend occurs because higher energy photons tend to penetrate through the glasses with greater ease than lower energy photons, requiring a greater thickness to attenuate the same quantity of radiation. Additionally, we can investigate the influence of the amount of Bi_2_O_3_ on the HVL values. Apparently, the HVL values decrease as the Bi_2_O_3_ content in the glasses increases. This can be explained according to the relation between HVL of the medium and its density. As the density of the glass is increased with the addition of Bi_2_O_3_, then more photons will interact with the dense sample, so we need a relatively thin glass to attenuate half of the incoming photons. Therefore, the HVL is decreased with the addition of Bi_2_O_3_ (or with increase in the density). In other words, BCrBi-0 has the greatest HVL at all tested energies (and the lowest Bi_2_O_3_ content), while the BCrCi-25 glass has the smallest HVL (and the greatest Bi_2_O_3_ content). For example, at 0.0810 MeV, the HVL values are 1.720, 1.018, 0.706, 0.531, 0.418, and 0.340 cm for BCrBi-0, BCrBi-5, BCrBi-10, BCrBi-15, and BCrBi-20, respectively, while at 0.9641 MeV, they are equal to 4.426, 4.231, 4.038, 3.849, 3.663, and 3.479 cm for the same respective glasses. Thus, since the BCrBi-25 has the greatest Bi_2_O_3_ content, this sample can be said to be the most space-efficient out of the investigated glass samples.

**Figure 10:**
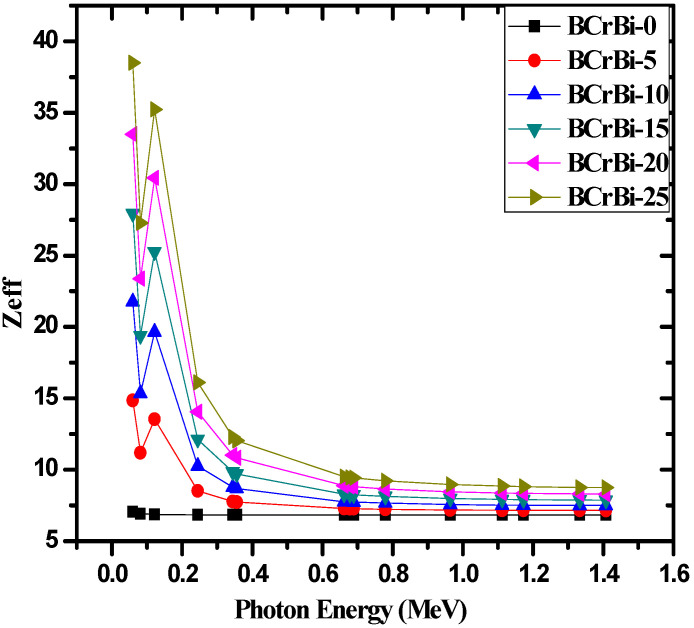
The effective atomic number (*Z_eff_*) of the BCrBiX glasses.

Figure 10 demonstrates the effective atomic number (*Z_eff_*) of the glasses against energy. The BCrBi-0 glass can be seen to have much smaller *Z_eff_* than the other samples due to the lack of Bi—which has a relatively large atomic number—in its composition. In BCrBi-0, we can see that *Z_eff_* is almost constant for this glass, which can be explained according to the chemical composition of this glass, since it contains only B_2_O_3_ and Cr_2_O_3_, and the atomic numbers of these elements are close to each other. The same trend for *Z_eff_* with energy was reported for other materials that contain different elements with close atomic numbers. For example, El-Kateb et al. reported the same trend in *Z_eff_* for some alloys, such as steel, bronze, and brass [30]. Additionally, Kaçal et al. [31] reported *Z_eff_* values for some polymers in the same energies used in this work and they found that the polymers which consist of H, C, O, and N have constant *Z_eff_*.

Additionally, the increase in Bi_2_O_3_ increases the *Z_eff_* of the glasses, which explains why the BCrBi-25 sample has the highest *Z_eff_* out of the investigated glasses. As the energy increases, *Z_eff_* can be observed to sharply decrease at first, and then slows down in its descent. The only exception is around 0.1 MeV, where a sharp spike in values occurs due to the k-absorption edge of Bi (which is why the BCrBi-0 sample does not have this spike). The sharp decrease in the *Z_eff_* values is due to the dominance of the photoelectric effect at low energies, while the slowed descent can be attributed to the Compton scattering interaction that dominates in the moderate energy range. This figure once again reaffirms the conclusion that the BCrBi-25 sample has the greatest potential for radiation shielding applications.

**Figure 11:**
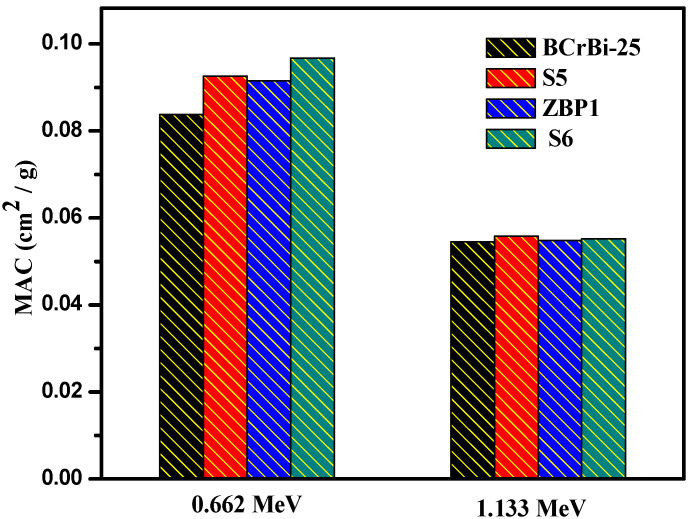
The MAC of BCrBi-25 glass sample compared with other commercial glasses.

**Figure 12:**
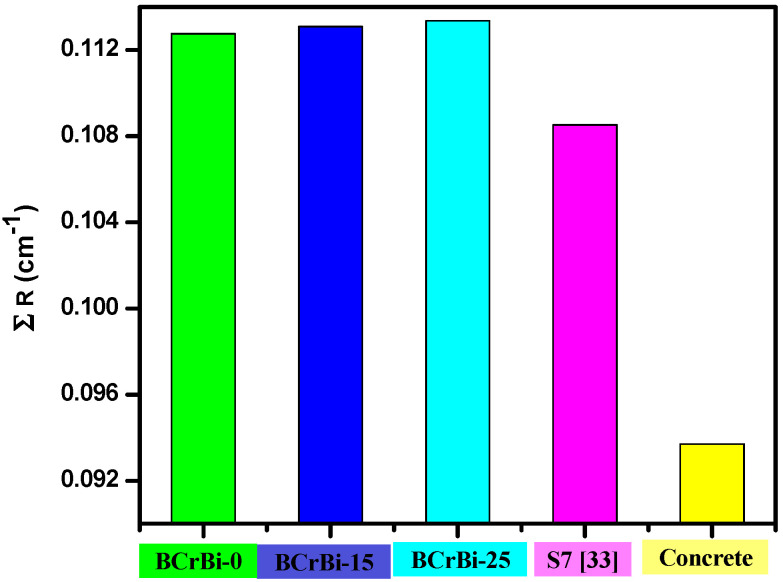
The effective removal for fast neutrons of the present glass samples compared with lead borate glass and concrete.

The MAC of the last present sample (BCrBi-25) was compared with different lead borate glasses, as shown in Figure 11, like S5 (50B_2_O_3_ + 10BaO + 10 Bi_2_O_3_ + 10CdO + 20PbO) [32], ZBP1 (10ZnO + 40 B_2_O_3_ + 50PbO) [33], and S6 (40B_2_O_3_ + 25Li_2_O + 35PbO) [34]. The results were 0.0838, 0.0926, 0.1002, and 0.0968 cm^2^/g for BCrBi-25, S5, ZBP1, and S6 at 0.662 MeV, respectively. However, at 1.332 MeV, MAC is in the same order of 0.0545, 0.0558, 0.0554, 0.0552 cm^2^/g. The results indicated that the present prepared samples (bismuth borate glasses) have a high attenuation, very close to commercial lead borate glasses. On the other hand, the samples are environmentally friendly and the cost is lower than other glass. Additionally, this glass is perfect for neutron shielding, whereas the determined fast neutron removable cross section (∑R) for the present sample is shown to have a remarkable superiority to the bismuth borate glass samples, compared with lead borate glass and concrete. In Figure 12, the values of ∑R are 0.1127, 0.1131, 0.1134, 0.1061, and 0.0937cm^−1^ for BCrBi-0, BCrBi-15, BCrBi-25, lead borate glass [35] and concrete [20], respectively.

## 4. Conclusions

The MAC of the (99-*x*)B_2_O_3_ + 1Cr_2_O_3_ + (*x*)Bi_2_O_3_ glass samples was measured through a narrow beam technique using a NaI(Tl) scintillation detector. The experimental data were compared with the XCOM data to confirm the accuracy in the narrow beam technique used in this work. The relative deviation between both techniques is less than 3%, which signifies that the experimental data can adequately be used to evaluate the shielding ability of the glasses. The results showed that TF is small at low energies and increases as the energy increases, and the addition of Bi_2_O_3_ leads to a reduction in the TF values. BCrBi-25 has the lowest transmission factor due to the high amount of Bi_2_O_3_ in its composition. At energy of 0.0810 MeV, BCrBi-25 has TF of 0.130, while at 1.1120 MeV, TF is equal to 0.833. The HVL data demonstrated that higher energy photons tend to penetrate through the glasses with greater ease than lower energy photons. The BCrBi-0 glass was found to have a much smaller *Z_eff_* than the other samples due to the lack of Bi in its composition. Additionally, the *Z_eff_* results showed that increasing the Bi_2_O_3_ causes an increase in the *Z_eff_* of the glasses, and thus the BCrBi-25 sample has the highest *Z_eff_* out of the investigated glasses. The different parameters presented in this study reaffirm the conclusion that the BCrBi-25 sample has the greatest potential for radiation shielding applications. A comparison of MAC for the prepared glasses with similar glasses (lead borate glasses) demonstrated that the present samples (bismuth borate glasses) have high attenuation that is very close to commercial lead borate glasses. The prepared glasses are less toxic than the lead borate glasses. Accordingly, we can conclude that the bismuth borate glasses presented in this work are promising radiation shielding materials due to their high attenuation performance, as well as their low cost and reduced toxicity.

## Figures and Tables

**Table 1:** Chemical composition and density of BCrBi glass samples.

Glass Code	Glass Composition (wt %)			Density (g/cm^3^)
	B_2_O_3_	Cr_2_O_3_	Bi_2_O_3_	
BCrBi-0	99	1	0	2.473
BCrBi-5	94	1	5	2.566
BCrBi-10	89	1	10	2.667
BCrBi-15	84	1	15	2.776
BCrBi-20	79	1	20	2.894
BCrBi-25	74	1	25	3.023

**Table 2:** The specification of the present point sources as well as its figures.

PTB Nuclide	Energy keV	Emission Probability	Activity kBq	Uncertainty kBq
Am-241	59.52	35.9	259	±2.6
Cs-137	661.66	84.99	385	±4.0
Eu-152	121.78	28.37	290	±4.0
	244.69	7.53		
	344.28	26.57		
	778.91	12.97		
	964.13	14.63		
	1112.0	13.54		
	1408.1	20.85		
Ba-133	80.99	32.9	275	±1.6
	356.01	62.05		

**Table 3:** The measured and theoretical mass attenuation coefficient (cm^2^.g^−1^) for the BCrBi glass samples.

Energy (MeV)	BCrBi-0			BCrBi-5		
	XCOM	Exp	dev (%)	XCOM	Exp	dev (%)
0.0595	0.1868	0.1838 ± 0.009	1.62	0.4181	0.4157 ± 0.021	0.58
0.0810	0.1630	0.1621 ± 0.015	0.54	0.2654	0.2680 ± 0.016	−0.98
0.1218	0.1419	0.1407 ± 0.011	0.84	0.2924	0.2879 ± 0.010	1.55
0.2447	0.1125	0.1142 ± 0.008	−1.5	0.1365	0.1348 ± 0.021	1.22
0.3443	0.0991	0.1004 ± 0.009	−1.35	0.1088	0.1094 ± 0.024	−0.57
0.3560	0.0978	0.0998 ± 0.014	−2.05	0.1067	0.1088 ± 0.017	−2.01
0.6617	0.0754	0.0741 ± 0.020	1.82	0.0771	0.0762 ± 0.011	1.22
0.7789	0.0700	0.0697 ± 0.017	0.52	0.0711	0.0705 ± 0.026	0.87
0.9641	0.0633	0.0638 ± 0.021	−0.8	0.0638	0.0622 ± 0.014	2.58
1.1120	0.0590	0.0580 ± 0.011	1.66	0.0593	0.0585 ± 0.010	1.33
1.4080	0.0523	0.0516 ± 0.023	1.44	0.0524	0.0530 ± 0.009	−1.17
	**BCrBi-10**			**BCrBi-15**		
	**XCOM**	**Exp**	**dev (%)**	**XCOM**	**Exp**	**dev (%)**
0.0595	0.6494	0.6552 ± 0.008	−0.89	0.8807	0.8629 ± 0.012	2.02
0.0810	0.3679	0.3633 ± 0.013	1.25	0.4703	0.4645 ± 0.019	1.25
0.1218	0.4429	0.4352 ± 0.024	1.74	0.5934	0.5967 ± 0.009	−0.55
0.2447	0.1605	0.1622 ± 0.014	−1.05	0.1845	0.1826 ± 0.008	1.01
0.3443	0.1185	0.1172 ± 0.011	1.11	0.1283	0.1305 ± 0.010	−1.77
0.3560	0.1156	0.1149 ± 0.011	0.58	0.1245	0.1242 ± 0.024	0.25
0.6617	0.0788	0.0782 ± 0.018	0.74	0.0805	0.0797 ± 0.030	0.98
0.7789	0.0721	0.0715 ± 0.012	0.92	0.0732	0.0733 ± 0.017	−0.14
0.9641	0.0644	0.0649 ± 0.013	−0.85	0.0649	0.0639 ± 0.015	1.55
1.1120	0.0596	0.0597 ± 0.018	−0.11	0.0599	0.0600 ± 0.014	−0.11
1.4080	0.0525	0.0520 ± 0.010	1.02	0.0527	0.0521 ± 0.017	1.12
	**BCrBi-20**			**BCrBi-25**		
	**XCOM**	**Exp**	**dev (%)**	**XCOM**	**Exp**	**dev (%)**
0.0595	1.1120	1.0960 ± 0.008	1.44	1.3433	1.3551 ± 0.024	−0.88
0.0810	0.5728	0.5817 ± 0.014	−1.55	0.6753	0.6670 ± 0.012	1.22
0.1218	0.7439	0.7272 ± 0.010	2.25	0.8944	0.9073 ± 0.028	−1.44
0.2447	0.2085	0.2055 ± 0.011	1.44	0.2325	0.2348 ± 0.014	−0.98
0.3443	0.1380	0.1397 ± 0.019	−1.22	0.1477	0.1508 ± 0.021	−2.08
0.3560	0.1334	0.1314 ± 0.012	1.55	0.1424	0.1403 ± 0.026	1.44
0.6617	0.0821	0.0808 ± 0.024	1.69	0.0838	0.0827 ± 0.017	1.36
0.7789	0.0742	0.0727 ± 0.025	2.01	0.0752	0.0741 ± 0.010	1.46
0.9641	0.0654	0.0649 ± 0.029	0.82	0.0659	0.0671 ± 0.012	−1.75
1.1120	0.0602	0.0601 ± 0.011	0.11	0.0605	0.0615 ± 0.010	−1.65
1.4080	0.0528	0.0524 ± 0.017	0.77	0.0529	0.0524 ± 0.009	0.88

## Data Availability

The data presented in this study are available on request from the corresponding author.
